# Can the TLR-4-Mediated Signaling Pathway Be “A Key Inflammatory Promoter for Sporadic TAA”?

**DOI:** 10.1155/2014/349476

**Published:** 2014-07-10

**Authors:** Giovanni Ruvolo, Calogera Pisano, Giuseppina Candore, Domenico Lio, Cesira Palmeri, Emiliano Maresi, Carmela R. Balistreri

**Affiliations:** ^1^Unit of Cardiac Surgery, Department of Surgery and Oncology, University of Palermo, 90127 Palermo, Italy; ^2^Department of Pathobiology and Medical and Forensic Biotechnologies, University of Palermo, Corso Tukory 211, 90134 Palermo, Italy; ^3^Department of Pathologic Anatomy, University of Palermo, 90127 Palermo, Italy

## Abstract

Thoracic aorta shows with advancing age various changes and a progressive deterioration in structure and function. As a result, vascular remodeling (VR) and medial degeneration (MD) occur as pathological entities responsible principally for the sporadic TAA onset. Little is known about their genetic, molecular, and cellular mechanisms. Recent evidence is proposing the strong role of a chronic immune/inflammatory process in their evocation and progression. Thus, we evaluated the potential role of Toll like receptor- (TLR-) 4-mediated signaling pathway and its polymorphisms in sporadic TAA. Genetic, immunohistochemical, and biochemical analyses were assessed. Interestingly, the rs4986790 TLR4 polymorphism confers a higher susceptibility for sporadic TAA (OR = 14.4, *P* = 0.0008) and it represents, together with rs1799752 ACE, rs3918242 MMP-9, and rs2285053 MMP-2 SNPs, an independent sporadic TAA risk factor. In consistency with these data, a significant association was observed between their combined risk genotype and sporadic TAA. Cases bearing this risk genotype showed higher systemic inflammatory mediator levels, significant inflammatory/immune infiltrate, a typical MD phenotype, lower telomere length, and positive correlations with histopatological abnormalities, hypertension, smoking, and ageing. Thus, TLR4 pathway should seem to have a key role in sporadic TAA. It might represent a potential useful tool for preventing and monitoring sporadic TAA and developing personalized treatments.

## 1. Introduction 

Heart and vascular system, including particularly the large elastic arteries, that is, the aorta, shows with advancing age a multitude of changes at different structural and functional levels [1–5]. As a result, vascular remodeling (VR) and medial degeneration (MD) occur [2, 4]. At the macroscopic level, these pathological entities induce weakening of aorta wall and a progressive stiffness [2, 4]. Endothelial dysfunction, increased oxidative stress, inflammatory reaction, inflammatory cell infiltration in aortic wall, apoptosis of vascular smooth muscle cells (VSMC)s, degeneration of aortic media, and elastin fragmentation and degradation represent their microscopic alterations [2, 4]. In turn, they can degenerate in aortic dilatation and aneurysm and increase the onset risk for complications, that is, aortic dissection and rupture. VR and MD are the typical pathological entities of several aorta diseases, including the inherited syndromic and familial forms of thoracic aortic aneurysm (TAA) and the sporadic forms [6]. Among these, sporadic TAA is becoming a common and serious health risk because of growing enhance of old people in Western populations [7, 8]. Aged population shows an increased incidence for sporadic TAA with advancing of years, as recently reported by epidemiological studies executed in geographic regions with stable populations with little out- or in-migration, such as in Minnesota and Sweden [9, 10]. Another determining factor related to the population ageing is the increased number of hypertensive individuals [11]. Hypertension is, indeed, a widely prevalent and important risk factor for cardiovascular diseases, including sporadic TAA, as established by recent guidelines [11].

Sporadic TAA is considered a pathology by unclear mechanisms [12]. However, current research's interest is enormously increasing, even if the literature data about its genetic, molecular, and cellular mechanisms are inconsistent. In addition, it is growing the opinion to consider thoracic aortic aneurysms, and particularly the sporadic forms, as immune/inflammatory diseases with a strong genetic component [13]. An active participation of both innate/inflammatory and clonotypic responses has been evidenced. He and colleagues observed an infiltration of inflammatory/immune cells in the media and adventitia from aorta samples of patients with sporadic TAA [14, 15]. Accordingly, we detected a significant high number of CD3+CD4+CD8+CD68+CD20+ cells in tissue aorta samples from patients with Stanford type A aortic dissection (TAAD). A significant role of inflammatory variants in the TAAD risk was also identified in our study [16]. Thus, chronic inflammation might contribute to the pathogenesis of sporadic TAA. This also leads to supposing that sporadic TAA may be the result of a complex combination of factors, including a high genetic propensity, epidemiology factors, age-related vascular alterations, hemodynamic stress, chronic inflammation, and aortic injury. In this complex scenario, the identification of the pathways activated by these chronic stressors might be crucial, in order to translate experimental data in clinical new personalized measures of prevention, diagnosis, treatments, and management.

In consistency with this suggestion, we propose that sporadic TAA might be the consequence of an active stimulation of a particular inflammatory signaling pathway, the Toll-like receptor (TLR)-4-mediated signaling pathway, able to recognize both pathogens and endogenous ligands. An increasing number of studies underlines the weight of TLR4-mediated signaling pathway in several cardiovascular diseases (CVD)s [23, 30–36]. In addition, its strong role in age-related aorta dysfunction, aneurysm's onset, and its complications (dissection or rupture) recently is emerging. A histopathological study demonstrated the TLR4 expression's profile in all cells of arterial wall, and particularly in endothelial cells (EC)s and VSMCs. It also evidenced its functional importance in mediating physiological aorta homeostasis and maintaining of protection against pathogens and damaging cell molecules, as well as in inducting pathological aorta phenotypes [23]. Recent experimental investigations in animal and* ex vivo* models also emphasises its role in the vascular aorta alterations (VR and MD) and their complications, that is, sporadic TAA, by inducing or modulating increased expression and activation of endothelium dysfunction and remodeling aorta pathways (i.e., angiotensin converting enzyme (ACE), endothelial oxide nitric synthase (eNOs), and metalloproteinase (MMP) pathways) [37–42]. In addition, genetic variants of TLR4-mediated signaling pathway have been associated with the susceptibility for several CVDs [30–36]. In particular, polymorphisms of TLR4 gene (NM-138554.1), and particularly the rs4986790 TLR4 polymorphism, have been associated with the risk for several CVDs and other age-related diseases (i.e., Alzheimer disease, prostate cancer, and diabetes), even if contrasting results have been reported [30, 32, 36, 43].

In the light of this current evidence, we sought in the present study to investigate the potential role of TLR4-mediated signaling pathway (promoter) in sporadic TAA. Precisely, we analysed the weight of ten genetic variants related to TLR4-mediated signaling pathway in disease susceptibility, evocation of aorta abnormalities, systemic inflammation (arterial-associated senescence secretor phenotype-AASSP), and vascular biological ageing. Thus, associations between their combined risk genotypes and typical pathological tissue phenotypes, systemic inflammatory mediator levels, inflammatory/immune infiltrate, apoptosis of VSMC cells, tissue MMP-9 amounts, and attrition of telomeres were also evaluated.

## 2. Materials and Methods

### 2.1. Patient Population

Our study included 161 individuals (127 men (78%) and 34 (22%) women; mean age: 63 ± 10.7) from Western Sicily enrolled precisely from January 2004 to July 2008 at time of their admission to Cardiac Surgery Unit of Palermo University Hospital. They were affected by sporadic TAA and diagnosed through imaging technologies (i.e., ECHO, CT and MRI) and with localization essentially in ascending aorta (precisely in aortic sinus and tubular portion and sometimes only in tubular portion) and in aortic bulb or both (see Table 1). For the patient's selection, histopathological analyses and exclusion criteria for syndromic and familial forms (e.g., Marfan and Ehler's Danlos syndromes) and autoimmune connective tissue disorders were utilised. According to 2010 guidelines, elective or acute surgical treatment (using wheat operation, Bentall-De Bono and Tirone David surgical techniques, whenever possible) and consequent resection were performed after evaluation of aortic transverse diameter sizes [44, 45]. Their evaluations are reported in detail in the paragraph “Evaluation of aortic transverse diameter sizes” in Supplementary Material available online at http://dx.doi.org/10.1155/2014/349476. The mean of these values was reported in Table 1.

Medical histories pertinent to aortic disease were obtained from patient's medical records. Thus, demographic and clinical features, comorbidity conditions, and pharmacological treatments were collected (see Table 1).

### 2.2. Control Population

The control group consisted of 128 subjects (61 (47%) men and 67 (53%) women; mean age: 61.08 ± 5.83 years), belonging to the same ethnic group of patients, in order to include in the study a very homogenous population. Ethnicity was confirmed, since parents and grandparents of both patients and controls were born in Western Sicily. Controls were in good health according to their clinical history and blood tests (complete blood cell count, erythrocyte sedimentation rate, glucose, urea nitrogen, creatinine, electrolytes, C reactive protein, liver function tests, iron, and proteins) (see Table 1). Their demographic and clinical features, comorbidity conditions, and pharmacological treatments were collected (see Table 1). Furthermore, echocardiography imaging examinations confirmed absence of any aorta wall histopatological abnormalities in all controls.

In order to detect histopathological abnormalities, control ascending aortas were obtained from 30 individuals (20 men and 10 women, mean age: 63.9 ± 10.3) who died for causes unrelated to aortic disease and having no sepsis at death time, as confirmed by autopsy (see Table 1).

Our study received approval from local ethic committees and all participants gave their informed consent. Data were encoded to ensure patient and control protection. All measurements were performed without knowledge about nature of material.

### 2.3. Histopathological Assays and Identification of MD Phenotypes

**Figure 1:**
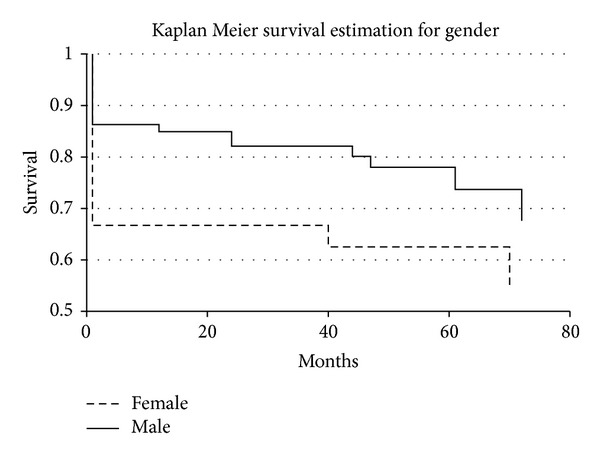
Survival in female and male patients after surgery.

**Figure 2:**
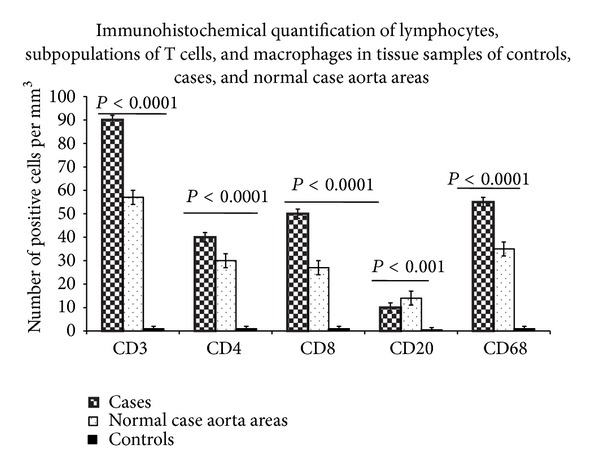
Morphometric quantification of lymphocytes, T cell subpopulations, and macrophages in tissue samples of the control aortas and patients and normal aorta case areas. CD3, CD4, CD8, CD20, and CD68 positive cells in media and adventitia and in 10 contiguous high-power fields (magnification 400x) were counted by two independent observers. Significant increased amounts of CD3+CD4+CD8+CD68+CD20+ cells were observed by comparing their values among the three groups (by ANOVA test). In particular, cases showed significant higher numbers of these cells than controls and normal aorta case areas.

Histopathological investigations were performed only in 100 aorta samples obtained from aortic wall of patients who underwent surgical repair, since some patient's aortas showed unsuitable histological conditions. The procedures used were previously reported in our recent studies [16, 46–49] and briefly described in online Supplementary Material. In addition, the following histological features were evaluated: (1) fibrosis (defined as an increase in interstitial collagen); (2) medionecrosis (defined as a focal loss of smooth muscle cell nuclei in the media); (3) cystic medial necrosis (defined as mucoid material accumulation); (4) focal or plurifocal medial apoptosis; (5) elastic fragmentation (defined as focal fragmentation of elastic lamellae in the media); (6) amounts of MMP-9; (7) inflammatory/immune cell infiltration. Histopathological abnormalities of aortic wall were graded and defined according to the definitions and grading systems used by Bechtel and colleagues [50] (see details reported in the section of online Supplementary Materials and precisely in Figures 1(S) and 2(S).) and previously described in our recent studies [16, 46–49].

In addition, typical phenotypes were detected as reported in online Supplementary Material and previously described in our recent study [49].

### 2.4. Immunohistochemical Assays

Immunohistochemical analyses were performed on aorta sections. Specific monoclonal antibodies were used, and standard techniques were performed as described in online Supplementary Material.

### 2.5. Tunel Testing

For detecting apoptosis, a TdT- (Terminal deoxynucleotidyl Transferase-) mediated X-dUTP (deoxyuridine triphosphate nucleotides) nick end-labeling (TUNEL) reaction (“In situ cell death detection kit”, Roche Diagnostics S.p.A, Milano, Italy) was used and performed as reported in detail in online Supplementary Material. Three apoptosis patterns were identified: absent, focal, and plurifocal medial apoptosis, as evidenced in Figure 2(S) in online Supplementary Material.

### 2.6. Semiquantitative Evaluation of MMP-9 by Immunohistochemical Assays

A semiquantitative evaluation of MMP-9 amount in aortic specimens was performed. Staining was classified as low, moderate, or high amount, as reported in Figure 2(S) in online Supplementary Material.

### 2.7. DNA Samples and Molecular Typing

DNA samples were obtained by blood samples from case and control individuals (see online Supplementary Material). They were genotyped for ten SNPs located in promoter and coding regions of selected candidate genes codifying molecules related to TLR4-mediated signaling pathway and with biological effects able to modulate the susceptibility for several CVDs, such as sporadic TAA. Information about these SNPs was acquired from dbSNP NCBI, the ENSEMBL database (http://www.ensembl.org/index.html), and the UCSC Genome Browser website (http://genome.ucsc.edu) and reported in Table S1 of online Supplementary Material. Genotyping was performed using the procedures illustrated in online Supplementary Material.

### 2.8. Inflammatory Plasma Molecule Measurements

Plasma IL-6, TNF-*α*, MMP-2, MMP-9, and CRP levels were measured according to method reported in online Supplementary Material.

### 2.9. Assessment of Mean Terminal Restriction Fragment Length

A marker of telomere leukocyte length was determined using DNA samples of 30 cases and 30 controls selected randomly, but having the same age and gender. The procedure used was previously described in the study of Balistreri and colleagues [51].

### 2.10. Statistical Analysis

All analyses were performed with R and Microsoft Excel software. Significant differences among qualitative variables were calculated by using Pearson *χ* ^2^ test. To analyze significant relationships among quantitative variables, Wilcoxon rank sum test was employed. Furthermore, odds ratios (OR) with 95% confidence intervals (CI) and their significance were calculated. To study mortality between male and female patients, Kaplan-Meier survival functions were calculated. Differences between survival curves with a Gehan test and appropriate Peto & Peto modification were carried out.

Allele and genotype frequencies were evaluated by gene count. Data were tested for goodness of fit between observed and expected genotype frequencies according to Hardy-Weinberg equilibrium, by *χ* ^2^ tests. Significant differences in frequencies among groups were calculated by using *χ* ^2^ test and appropriate tables (2 × 2 and 3 × 3 tables, etc., were appropriate and corrected by Bonferroni). Significant relationship between genetic variables and pathology risk was analysed using quasi-likely hood binomial models.

Analysis of variance (ANOVA) test (corrected by Bonferroni) was also utilised to compare positive inflammatory/immune cells between case and control aorta samples. Unpaired *t*-test (Welch corrected) was, utilised to analyse positive inflammatory/immune cells between pathological and normal case aortas. To identify possible correlations between CD3+CD4+CD8+CD68+ cell number and aorta aneurysm diameter, nonparametrical Spearman correlation test was also used. The same test was utilised to evaluate the correlations between the severity of all examined histopathological abnormalities and patient features or the major risk factors (i.e., age, gender, aortic diameter, hypertension, diabetes, and smoking) and to assess the correlations between the MMP-9 and severity of elastic fragmentation.

Quantitative values of the cytokines, MMPs and CRP, were expressed as mean ± SD. To assess their differences, unpaired *t*-test (Welch corrected) was utilised. Categorical variables were compared by chi-square test or Fisher's exact test. Their correlations were assessed using Spearman's rank correlation. A *P* < 0.05 was considered statistically significant.

The difference in mean TRF length between cases and controls was analysed using an independent sample “*t*” test. The difference in mean TRF length in subjects with different risk factors was analysed using bivariate correlation for continuous variables and independent samples “*t*” test for categorical variable. The independent effect of age, sex, and other risk factors on the mean TRF length was analysed using a linear regression model controlling for case control status.

## 3. Results

### 3.1. Patient and Control Characteristics

We analyzed the patient and control features summarized in Table 1. The elevated number of sporadic TAA men (127 versus 34 women) led us to compare all patient features according to gender. No statistical significant differences were detected, with exception of smoking (67 male versus 6 female, *P* < 0.001 by Pearson *χ* ^2^ test, 2 × 2 table; OR 5.16 (1.92–16.32), *P* = 0.0002 by Fisher test). However, among the major risk factors, hypertension characterised 79% of all patients, opportunely treated with medications like ACE inhibitors and beta-blocker, and so forth, during the follow-up and after surgery (see Table 1). According to gender, no significant differences were also observed in evaluating long-term survival (80 months) after surgery. However, OR was higher for women (4.5 (3.2–6.9) versus 2.6 (2.2–2.9) for men). Significant differences were, detected in short-term survival (30 days). Compared with male patients, females showed increased 30 day mortality: 9 of 34 female (26%) versus 10 of 127 male (8%) (*P* = 0.049 by Gehan test; OR 6.5 (1.5–6.8), *P* = 0.01 by Fisher test) (see Figure 1). These data correlated with several clinical conditions observed in the major female number after surgical repair (data not shown). Comparisons between patient and control features were also assessed. No statistical significant differences were detected, with exception of hypertension (127 versus 40 *P* < 0.001 by Pearson *χ* ^2^ test, 2 × 2 table; see Table 1).

### 3.2. Allele Frequencies of the Ten TLR4 Related Pathway SNPs and Identification of a Combined Risk Genotype Related to TLR4-Mediated Signaling Pathway

In order to demonstrate the key role of TLR4-mediated signaling pathway in the pathophysiology of sporadic TAA and to support our hypothesis, we compared the allele frequencies of ten selected SNPs between 161 patients and 128 controls. Significant differences were only found for the following SNPs: rs4986790 TLR4, rs333 CCR5, rs2070744 eNOs, rs1799752 ACE, rs3918242 MMP-9, and rs2285053 MMP-2 (see Table 2). Among these, we interestingly observed that the rs4986790 TLR4 polymorphism confers a higher susceptibility for sporadic TAA (OR = 14.4, *P* = 0.0008) (see Table 2). The protective +896 G TLR4 allele associated with a low risk of age-related diseases [32] has a frequency only of 0.3% (1) in cases versus 4% (11) in controls (see Table 2). In addition, using a quasi-hood binomial statistical model, we obtained that the rs4986790 TLR4, rs1799752 ACE, rs3918242 MMP-9, and rs2285053 MMP-2 SNPs are independent risk factors for sporadic TAA (*P* = 0.001). Considering the biological effects of these SNPs (see Table S1), we assessed the frequency of +896ATLR4/DACE/−1562TMMP-9/−735TMMP-2 risk genotype in cases and controls. By comparing it with frequency of other combinations, combined risk genotype, “*high responder genotype,*” was significantly represented in cases than controls. Indeed, 46 patients were carriers of the combined risk genotype versus 10 controls (*P* = 0.000009, by *χ* ^2^ test; OR = 4.7, *P* < 0.0001 by Fisher's exact test, see Table 3).

### 3.3. Evaluation of the Role of +896ATLR4/DACE/−1562TMMP-9/−735TMMP-2 Combined Risk Genotype in Influencing the Levels of Systemic Plasma Inflammatory Mediators

Given the overrepresentation of this combined risk genotype in patients and its strong role in the susceptibility (OR = 4.7, see Table 3) for sporadic TAA, we evaluated its biological effect in influencing the grade of chronic inflammation. Thus, we assessed the eventual significant differences in systemic plasma levels (AASSP levels) of IL-6, TNF-*α*, CRP, and MMP-2 and -9 between all patients and all controls. The same analysis was performed in cases bearing combined risk genotype versus no case carriers and in case carriers versus control carriers. As reported in Table 4, we detected significant differences of all systemic plasma mediators between patients and controls. However, the very interesting and promising datum was the presence of higher levels of all mediators examined in cases bearing combined risk genotype than both cases bearing other genotypes and control carriers of combined risk genotype, as illustrated clearly in Table 4. Furthermore, we detected that higher plasma levels of MMP-2 and -9 levels from cases bearing combined risk genotype significantly correlated with the moderate and elevated amounts of MMP-9 detected reciprocally from their tissue aorta samples (*r* = 0.397, *P* = 0.001; *r* = 0.234, *P* = 0.03 by nonparametrical Spearman correlation test; data not shown). In particular, cases having combined risk genotype showed a significant association between increased plasma MMP-9 and MMP-2 levels and elevated amounts of MMP-9 (*P* = 0.006 by *χ* ^2^ test; data not shown). Consistent with these data, positive correlations were detected in the cases with combined risk genotype between the increased plasma levels of MMP-9 and MMP-2 and elastic fragmentation and the elevated amounts of MMP-9 observed in their tissue aorta samples (*r* = 0.497, *P* = 0.0001; *r* = 0.267, *P* = 0.03, *r* = 0.342, *P* = 0.006, resp., by nonparametrical Spearman correlation test; data not shown).

### 3.4. Assessment of the Biological Effect of +896ATLR4/DACE/−1562TMMP-9/−735TMMP-2 Combined Risk Genotype, “High Responder Genotype,” and Alleles of TLR4 Gene in the Healthy Control Group

In verifying the influence and the biological effect of combined risk genotype on levels of systemic inflammatory mediators, and consequently its role in the occurrence of VR, MD and their complications, such as sporadic TAA, their quantities were compared in control carriers versus no control carriers (10 versus 118, as reported in Table 3). As shown in Table 5, this comparison demonstrated significant differences of all systemic inflammatory mediators (IL-6, TNF-*α*, CRP, and MMP-2 and -9) in controls bearing the high responder genotype than those with other genotypes. Thus, combined risk genotype seems to mediate a crucial biological effect on the levels of systemic inflammatory mediators and, hence, in the evocation of MD, VR, and sporadic TAA.

We also evaluated the potential significant differences of levels of systemic AASSP mediators in controls screened only for the presence of rs4986790 TLR4 polymorphism. Controls bearing the allele +896G, associated with a blunted innate/inflammatory response, showed significant reduced levels of all systemic AASSP mediators than carriers of the +896A TLR4 allele (see Table 5). Thus, controls with the TLR4 proinflammatory +896A allele had significant levels of systemic mediators, but their magnitude was lower than that observed in controls with the combined risk genotype.

### 3.5. Detection of the Inflammatory Cell Infiltration in Tissue Aorta Samples from Patients and Controls and the Examination of the Role Mediated of the Combined Risk Genotype

Furthermore, infiltration of lymphocytes and macrophages was also detected in tissue aorta wall samples from patients, control aortas, and normal areas from the same TAA tissues. A significant higher infiltrate of lymphocytes and macrophages in tissue aorta wall samples from patients compared with both control aortas and normal areas from the same TAA tissues was observed (see Figure 2). Interestingly, the infiltration of inflammatory/immune cells was particularly considerable in the vasa vasorum of adventitia from the aorta patient samples. In contrast, a very small infiltrate of these cells was observed in control aortas, which appears to be less significant in respect to that found in normal aorta TAA areas. CD20+ cell infiltrate was less represented in three groups, even if significant differences were observed by comparing the three cohort tissues (see Figure 2). Immunostaining with CD68 antibody also indicated that macrophages were prevalently present in aortas from TAA patients in respect to control aortas and normal areas from the same TAA tissues (see Figure 2).

**Figure 3:**
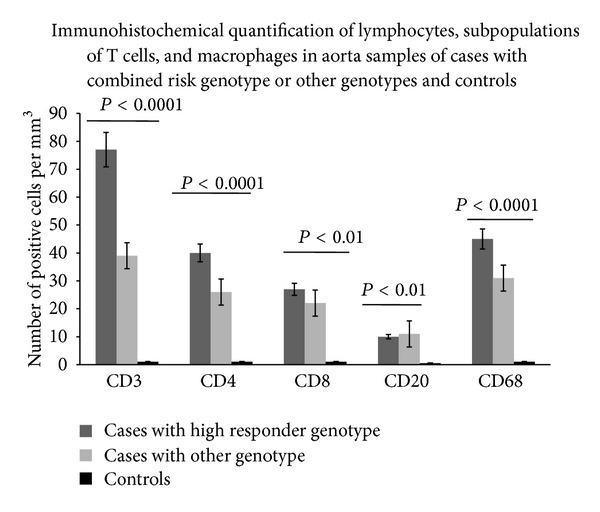
Morphometric quantification of lymphocytes, T cell subpopulations, and macrophages in aorta samples of cases with high responder genotype, other genotypes, and controls. CD3, CD4, CD8, CD20, and CD68 positive cells in media and adventitia and in 10 contiguous high-power fields (magnification 400x) were counted by two independent observers. Significant increased amounts of CD3+CD4+CD8+CD68+CD20+ cells were observed by comparing their values among the three groups (by ANOVA test). In particular, cases with high responder genotype had higher numbers of these cells than controls and cases with other genotypes.

In order to validate the biological effect mediated of combined risk genotype on the levels of systemic plasma inflammatory mediators, we also compared inflammatory/immune infiltrate between patients bearing high responder genotype and those with other genotypes. According to encouraging data obtained on levels of systemic plasma inflammatory mediators, we assessed a higher inflammatory/immune infiltrate in tissue aorta samples from patients bearing high responder genotype than those bearing other genotypes and control aortas (see Figure 3). Positive correlation was identified between the number of CD3+CD4+CD8+ CD68+ cells observed in aorta samples from patients bearing high responder genotype and the histological abnormalities observed through histopathological and immunohistochemical assays and Tunel testing (see Table 6). As reported in Table 6, the number of CD3+CD4+CD8+ CD68+ cells also correlated with the increased plasma levels of IL-6, TNF-*α*, CRP, and MMP-2 and -9.

### 3.6. Identification of Typical MD Phenotypes and Their Potential Association with Combined Risk Genotype

In addition, the 46 patients with combined risk genotype showed for the 89% a typical morphological aorta's phenotype, defined in our previous study as phenotype III, characterized by elevated cystic MD, plurifocal medial apoptosis, and increased MMP-9 amount (as reported in online Supplementary Material) [42]. Positive correlations were identified between the severity of histopatological abnormalities characterising this phenotype and hypertension, smoking, and age (*r* = 0.179, *P* = 0.03; *r* = 0.345, *P* = 0.001; *r* = 0.267, *P* = 0.02, resp., by nonparametrical Spearman correlation test; data not shown).

### 3.7. Assessment of the Weight of Combined Risk Genotype in Inducing Vascular Biological Ageing: Analysis of the Gold Marker of Biological Ageing “Telomere Attrition”

Consistent with these interesting data, we also evaluated whether the combined risk genotype can likely have a strong role in determining vascular senescence and onset of sporadic TAA. To this purpose, we examined the mean of blood leukocyte telomere length using terminal restriction fragment assay (TRF test, a southern blot technique) and blood samples from 30 patients and 30 controls, using a procedure described in two of our recent studies [51]. Thus, we detected that the case group had a mean TRF length (4.675 ± 0.605 kbp, data not shown), significantly lower than that observed in the control group (6.218 ± 0.485 kbp, data not shown). A difference of 1.543 bp was observed between cases and controls (95% confidence interval 68 bp to 201 bp, *P* = 0.001). There was no significant change in the mean TRF length with age in both patients and controls (6 bp decrease per year, SD = 4; *P* = 0.25). There was also no significant correlation between aneurysm size and mean TRF length (*P* = 0.46). There was no significant difference in the mean TRF length between male and female patients. Comparisons between mean TRF length and the risk TAA factors including gender, smoking, hypertension, diabetes, and family history were also evaluated. Sex as an independent risk factor did not have any significant effect on the mean TRF length (*P* = 0.76). In contrast, smoking and hypertension in S-TAA cases were the only risk factors which were significantly associated with the mean TRF length (*P* = 0.01). S-TAA subjects with smoking and hypertension history had a significantly shorter mean TRF length (4.491, SD = 0.237) compared to subjects without these risk factors (5.997, SD = 0.302), (mean difference 132 bp, confidence interval 11 bp to 245 bp, *P* = 0.01).

Stratifying the 30 patients having low mean TRF length for combined risk genotype, we interestingly observed that 85% (versus 8% for controls) were carriers of combined risk genotype (*P* = 0.001 by *χ* ^2^ test, 2 × 2 table; data not shown) and had significant higher levels of plasma inflammatory mediators (IL-6: 13.8 ± 2.3 versus 5.1 ± 1.4, *P* < 0.001; TNF-*α*: 15.5 ± 1.4 versus 8.2 ± 1.2, *P* < 0.001; CRP: 18.4 ± 1.3 versus 6.6 ± 2.8, *P* < 0.0001; MMP-9: 59.9 ± 2.8 versus 11.6 ± 1.8, *P* < 0.0001, and MMP-2: 57.8 ± 3.5 versus 13.7 ± 1.9, *P* < 0.0001, resp.; data not shown), increased amounts of CD3+CD4+CD8+CD68+CD20+ cells than controls and patients with other genotypes (*P* < 0.0001, by ANOVA test (corrected by Bonferroni)).

## 4. Discussion

Sporadic TAA is predominantly a silent ailment, until rupture or dissection occur, and insidious in its onset and progression. Its diagnosis is exclusively based on imaging technologies (i.e., ECHO, CT, or MRI) [12]. Accordingly, it is very crucial to early predict, diagnose, and treat sporadic TAA, characterised by lack of medical optional treatments and disease biomarkers, such as blood tests. Blood tests might consent to detect in general population individuals at sporadic TAA risk, to monitor its progression and predict complications. To this purpose, it should be crucial understanding cellular and molecular mechanisms and genetic risk factors. Recently, it has been suggested that aortic aneurysms, and particularly the sporadic forms, are immune diseases with a strong genetic component [13]. In particular, a particular involvement of chronic inflammation is emerging. We have evidenced this crucial aspect of the complex pathophysiology of this disease in our recent studies [16, 46–49]. In patients with TAAD, we observed high levels of immune/inflammatory cells and a significant association of some inflammatory polymorphisms with the TAAD susceptibility. In line with this, He and colleagues recently observed an increased immune/inflammatory infiltrate in aorta samples of patients with sporadic TAA [14, 15]. This is leading to identify the inflammatory pathways, which might operate as key link between the onset of sporadic TAA and immune system. Their recognition should be very imperative in order to translate experimental data in clinical new personalized measures of TAA prevention, diagnosis, treatments, and management.

Considerable and convincing evidence links the pathophysiology of atherosclerosis, cardiac dysfunction, congestive heart failure, and other vascular diseases with the TLR-4-mediated signaling pathway, as amply stressed by Frantz and colleagues [31]. Recently, the group of Pasterkamp also provided an overview of the endogenous molecules, released under cellular cardiovascular stress and damage, which can trigger innate immunity via TLR-4-mediated signaling pathway in CVDs [33]. In the specific case of sporadic TAA, recent experimental investigations in animal and* ex vivo* models also emphasise its role in the vascular aorta alterations (VR and MD) and their complications, such as sporadic TAA, by evocating or modulating increased expression and activation of endothelium dysfunction and remodeling aorta pathways [23, 37–42]. On the other hand, Pryshchep and colleagues demonstrated the TLR4-mediated signaling pathway expression in all cells of arterial wall and particularly in ECs and VSMCs. In addition, they also evidenced its functional importance in both mediating physiological aorta homeostasis and maintaining protection, as well as in inducting pathological aorta phenotypes, that is, VR and MD [23]. Furthermore, Song and colleagues demonstrated that signaling via TLR4-mediated signaling pathway and its signal adaptors, that is, MyD88, is responsible for the age-elevated basal IL-6 response using VSMCs from aged *TLR*4^−/−^ and *Myd*88^−/−^ mice [37]. Eissler and colleagues observed an increased hypertension-related expression of TLR4-mediated signalling pathway in vascular cells of untreated hypertensive rats [38]. The group of Golzales-Ramos underlined that circulating Heat Shock protein 70, associated with an increased cellular aorta's damage, regulates the profibrotic response of human aorta SMCs through increased transforming growth factor type-1 (TGF-1) expression, evocated by TLR4-mediated signaling pathway [39]. In addition, Li and colleagues reported the role of TLR4-mediated signaling pathway in regulating the MMP-9 expression in human VSMCs [40]. Bucci and colleagues recently emphasized as the vascular thoracic aorta homeostasis and its alteration in rats is based on the activity of TLR4-mediated signaling pathway and its cross talk with other stress and stretch pathways, that is, ACE, eNOs, and MMP pathways [41]. Furthermore, a recent study demonstrated in apolipoprotein E-deficient mice that it is possible to limit the inflammatory process by blocking TLR4/c-Jun N terminal kinase signaling pathway with Rosiglitazone in the initiation stages of aortic aneurysm development [42].

This encouraging and increasing evidence, fruit prevalently of animal investigations, and our recent data on TAAD [16, 46, 48], led us to analyze the potential role of genetic variants related to TLR4-mediated signaling pathway in the complex pathophysiology of sporadic TAA. Until now, no literature data exist about their role in sporadic TAA. Thus, our study represents the first report which, through a human* ex vivo* study approach, evidenced as some polymorphisms related to TLR4-mediated signaling pathway significantly modulate the sporadic TAA risk: rs4986790 TLR4, rs333 CCR5, rs2070744 eNOs, rs1799752 ACE, rs3918242 MMP-9, and rs2285053 MMP-2 polymorphisms. Among these, the rs4986790 (+896A>G) TLR4 polymorphism confers a higher susceptibility for sporadic TAA (OR = 14.4, *P* = 0.0008). However, it represents an independent risk TAA factor for sporadic TAA, as well as the rs1799752 ACE, rs3918242 MMP-9, and rs2285053 MMP-2 SNPs. Thus, we assessed whether their combined genotype constitutes a risk profile. A significant overrepresentation of their combined risk genotype (+896ATLR4/DACE/-1562TMMP-9/-735TMMP-2) was observed in cases than controls (46 versus 10, *P* < 0.000009) by comparing it with frequency of other combinations. In addition, it was associated a significant risk for sporadic TAA (OR = 4.7; *P* < 0.0001). Cases bearing combined risk genotype showed higher systemic inflammatory mediator levels than those with other genotypes and control carriers. In particular, they had higher plasma levels of MMP-9 and -2 which correlated with the amounts of MMP-9 and elastic fragmentation observed in their tissue aorta samples. A higher chronic inflammatory infiltrate was also found in cases bearing combined risk genotype, which positively correlated with histological abnormalities and levels of mediators. In addition, they showed in their tissue aorta samples a typical morphological phenotype, characterized by elevated cystic MD, plurifocal medial apoptosis, and increased MMP-9 amounts, and defined in a previous study as phenotype III [42]. Furthermore, we detected that combined risk genotype influences vascular biological ageing, evaluating the gold standard ageing marker, the telomere length, in a small number of cases and controls, selected randomly, but having the same age and gender. It characterized the 85% of the cases examined, which had lower telomere length, higher levels of mediators, increased amount of chronic inflammatory infiltrate. This results concord with the preliminary data reported in a previous study and the recent literature reports [51–53].

These interesting results led us to evaluate the biological effect of the combined risk genotype. Thus, we analysed the levels of systemic mediators in healthy group. Controls bearing high responder genotype showed higher levels of systemic mediators than control carriers of only the rs4986790 TLR4 polymorphism. These results are in agreement with our previous data [54, 55]. Indeed, in clarifying and confirming the biological effects of rs4986790 TLR4 polymorphism and its role in the pathophysiology of age-related diseases, including CVDs, Alzheimer disease, prostate cancer, diabetes, and longevity, we assessed the levels of IL-6, TNF-*α*, IL-10, and eicosanoids in LPS-stimulated whole blood samples* in vitro* of 50 young healthy Sicilians, screened for the presence of rs4986790 TLR4 and −765G>C PTGS2, −1708G>A 5-Lo polymorphisms [54]. Significantly higher levels of both proinflammatory cytokines and eicosanoids were observed in individuals bearing the rs4986790 TLR4 polymorphism. However, their magnitude significantly was more increased in individual's carriers of their combined genotype. In addition, significantly lower levels of inflammatory mediators were observed in carriers bearing the TLR4 mutation (the +896G allele), whereas the anti-inflammatory IL-10 values were higher [54]. The same results were detected in this study in the controls carriers of the +896G TLR4 allele. Thus, these data strengthen our suggestion, stressed for other age-related diseases including Alzheimer disease and prostate cancer, that polymorphisms related to TLR4-mediated signaling pathway may have a major influence on the pathophysiology of a disease, such as sporadic TAA, when they operate in combination to create a “risk profile” [56, 57].

## 5. Limitations and Conclusions 

In the complex, our data seem to suggest the strong relevance of TLR4-mediated signaling pathway in inducing MD, age related VR, and their complications, such as sporadic TAA. On the other hand, our findings emphasise as a combined risk genotype associated of TLR4-mediated signaling pathway are able to modulate the grade of aorta age-related phenotypical, histological, and systemic abnormalities and consequently vascular aorta ageing, onset, and progression of sporadic TAA. They also lead us to suggest that this signaling pathway might also be an optimal target for new therapeutic treatments able to retard or block the typical aorta age-related changes which determine endothelial dysfunction, MD, and VR. This might open new perspectives for the prevention of both aortic VR and MD and sporadic TAA, by using combined risk genotype (+896ATLR4/DACE/−1562TMMP-9/−735TMMP-2) as optimal genetic biomarker for the earlier detection of this silent pathology in preliminary phases and to treat with different and specific therapies depending on individual's genotypes. Consequently, it also leads to view vascular ageing as a modifiable risk factor, particularly for aortic disease. In the specific case of sporadic TAA, the therapeutic potential of TLR4-mediated signaling pathway might be defined through the use of its agonists or antagonists, whose effects have been prevalently experimented in mice, rats, and cultures as reported in literature [35]. This might consent to reduce chronic age-related inflammation and limit the dysfunction of ECs and endothelial progenitor cells (EPCs), which move towards injured endothelium or inflamed tissues and incorporate into foci of neovascularisation, thereby improving blood flow and tissue repair [58–60]. On the other hand, a recent investigation reports the involvement of TLR4-mediated signaling pathway in maintaining the stem cell phenotype of EPCs and enlarging this population [61]. This finding reveals a novel aspect of the multiple-faced TLR-4-mediated signaling pathway biology, and it may open new prospects for using TLR4 agonists in promoting the production of EPCs for clinical use. In addition, recent research underlines that miRNAs might play important roles in this scenario, modulating TLR-4-mediated signaling pathway activation [62]. MiRNAs seem, indeed, to have two opposite roles: TLR-4-mediated pathway activation and NF-*κ*B signaling inhibition, in a complex scenario where low and chronic inflammation prevails, likely also sustained by cell senescence secretome. MiRNAs inhibition effect probably belongs to the different levels of anti-inflammatory pathways which have evolved to abate TLR-4 signaling to prevent cell and aorta destruction [62].

Based on our findings, we also suggest another possible therapeutic intervention to apply in the preclinical phase in subject's carriers of combined high risk genotype to the aim to retard to limit the onset and progression of the vascular ageing and its complications, such as sporadic TAA. Precisely, we propose antibody-mediated stimulation of TAM receptors involved in the inhibition of the inflammatory response [29]. The sequential induction of this pathway and its integration with upstream TLR and cytokine signaling networks may impact the evocation of the release of inflammatory mediators limiting the inflammatory response and consent to modulate the telomere/telomerase system reducing the senescence of both the aorta wall cells and the EPCs able in repairing aorta injury [29]. On the other hand, patients with CVDs exhibit a reduced EPC number and function [63–65]. It has become increasingly apparent that these changes may be effected in response to enhanced oxidative stress, possibly as a result of systemic and localized inflammatory responses. Recent studies suggest that inflammation and oxidative stress modulate EPC bioactivity [63–65].

The weight of our findings and suggestions might be certainly implemented validating them in a larger sample size, even if our data are the result of a relatively small sample and a very homogenous population. In addition, gene expression analyses, immunohistochemical TLR4 quantification, and soluble TLR4 level detection represent further objectives of our future studies. They should consent to translate with major emphasis our promising data in personalized treatments of a pathology, the sporadic TAA, which clinically and predominantly is silent, until rupture or dissection occurs, and insidious in its onset and progression. Furthermore, until now its diagnosis is also exclusively based on imaging technologies.

**Figure 4:**
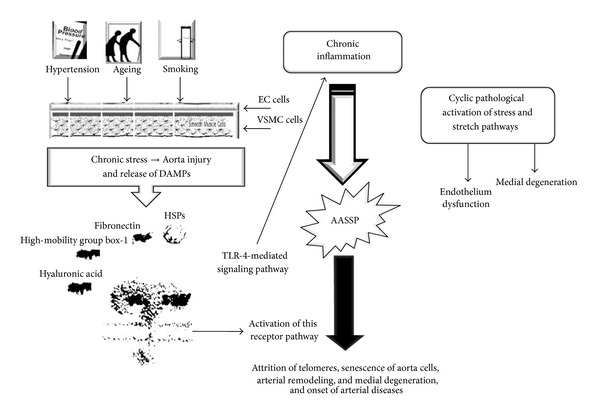
Our model about the pathophysiology of sporadic TAA, the* model of the pathway from the double-face.* The major risk factors, hypertension, age, and smoking, induce an increased production of reactive oxygen species (ROS) [17–19] and an upregulation of local renin-angiotensin system [16] and the tissue injury, initially involving ECs and subsequently VSMCs. This determines the release of some damage-related products and proteins (i.e., heart shock proteins (HSPs), high-mobility group box-1, low molecular hyaluronic acid, fibronectin fragments, and others), called danger-associated molecular patterns (DAMPs) [20]. DAMPs alert innate/inflammatory immune system interacting with TLR4 mediated signaling pathway, able to recognize both pathogens and endogenous ligands [21, 22]. Originally described as part of the first-line defense against Gram-negative bacteria, the best known member of TLRs, the TLR4, expressed on leukocytes and a large array of tissue and cell types, such as all aortic wall cells (particularly ECs and VSMCs), responds to these signals [23]. As a consequence, TLR4 activates and triggers an inflammatory response [24–28]. In turn, this determines a typical phenotypic switching of EC and VSMC cells due to activation of stress and stretch pathways accompanied by their dysfunction and senescence [27, 28]. In particular, it implies a differential change in their gene expression profile due prevalently of activation of Nuclear Factor-*κ*B (NF-*κ*B) transcription factor and followed by production and release of the so-called* arterial-associated senescence secretor phenotype* (AASSP) characterized by numerous inflammatory mediators, mitotic and trophic factors, proteoglycans and metalloproteinases (MMP)s, such as MMP-2 and -9, and vasoactive molecules [17, 24–29]. In addition, this also induces the reduction of nitric oxide (NO) [28]. This complex scenario results in modifications of vascular tone and permeability and degradation of components of extracellular matrix (ECM) and elastic fragmentation. VR and MD are, hence, evocated, which can evolve in aneurysm, dissection and rupture of aorta wall [17].

Finally, our obtained data led us to postulate a potential model about the pathophysiology of sporadic TAA, which might be defined as* model of the signaling pathway from the double-face, *given its features (see Figure 4). We foretell that it can lead several researchers to perform investigations focused to clear the complex puzzle of this pathology.

## Supplementary Material

In this section, we reported in detail the procedure used for evaluating aortic transverse diameter sizes. In addition, criteria, definitions and grading systems for tissue sample collection, staining, histopathological and immunohistochemical assessment, ELISA for quantifying inflammatory plasma molecules, Terminal deoxynucleotidyl transferase dUTP Nick End Labeling (TUNEL) testing for evaluating apoptosis, semiquantitative MMP-9 evaluation, assessment of mean terminal restriction fragment length and genotyping were also described in detail. Furthermore, we reported in Table 1S information about the 6 genes and 10 SNPs analysed and their Biological Effects. In Figures 1S and 2S we reported the control aortas and histo-pathological abnormalities in aorta tissues of S-TAA patients and medial apoptosis and MMP-9 amounts in tissue samples, reciprocally.

## Figures and Tables

**Table 1:** Demographic and clinical characteristics, comorbidity conditions, and pharmacological treatment of 161 patients affected by sporadic TAA, 128 control subjects, and 30 aorta controls.

Variables	Patients (*N* = 161)	Male (*N* = 127)	Female (*N* = 34)	Controls (*N* = 128)	*P*1 (cases versus controls)	*P*2 (male versus female)	Aorta Controls (*N* = 30)
Demographic characteristics							
Age, mean (SD)	63 (10.7)	63 (11)	64 (9)	61.1 (5.8)	0.834	0.594	63.9 (10.3)
Male sex, number (%)	127 (78)			61 (47)			
Female sex, number (%)	34 (22)			67 (53)			
Body mass index, mean (SD)	27 (4.3)	26.9 (3.8)	27.5 (5.6)	26.9 (2.9)	0.898	0.963	25.6 (2.9)
Size and location							
Size (mm), mean (SD)	53.3 (8)	52.9 (7.5)	55 (9.8)	0 (0)		0.191	0 (0)
Location, number (%):				0 (0)		0.198	
Ascending aorta	81 (50)	20 (59)	61 (48)				
Aortic Bulb	18 (11)	1 (3)	17 (13.4)				
Ascending aorta and Aortic bulb	62 (39)	13 (38)	49 (38.6)				
Comorbidity conditions, number (%)							
Cardiovascular Ischemic Familiarity	59 (36.6)	48 (38)	11 (32)	34 (27)	0.089	0.7	1 (3.3)
Smoking	73 (45)	67 (53)	6 (18)	66 (51)	0.351	**<0.001**	3 (10)
Hypertension	127 (78.9)	101 (80)	26 (76)	40 (31)	**<0.001**	0.879	2 (6.6)
Dislipidemy	37 (23)	30 (24)	7 (21)	20 (16)	0.158	0.886	0 (0)
Diabetes mellitus	24 (15)	16 (13)	8 (24)	16 (13)	0.677	0.187	0 (0)
Renal failure	5 (3.1)	4 (3.1)	1 (2.9)	0 (0)	0.168	0.621	0 (0)
Dissection	18 (11)	5 (15)	13 (10)	0 (0)		0.669	0 (0)
Aortic valve pathology, number (%):							0 (0)
Normal	90 (56)	71 (56)	20 (59)	0 (0)		0.766	0 (0)
Prolapse	21 (13)	17 (13)	3 (9)	0 (0)			0 (0)
Vascular calcium fibrosis	50 (31)	39 (31)	11 (32)	0 (0)			0 (0)
Aortic valve dysfunction, number (%):							
Normal	32 (20)	26 (20.4)	6 (17.6)	0 (0)		0.91	0 (0)
Faint incontinence	29 (18)	22 (17)	7 (20.4)	0 (0)			0 (0)
Moderate incontinence	34 (21)	28 (22)	6 (17.6)	0 (0)			0 (0)
Severe incontinence	44 (28)	35 (28)	9 (26.4)	0 (0)			0 (0)
Faint stenosis	1 (0.6)	1 (0.8)	0 (0)	0 (0)			0 (0)
Moderate stenosis	2 (1.2)	1 (0.8)	1 (3)	0 (0)			0 (0)
Severe stenosis	19 (11.2)	14 (11)	5 (15)	0 (0)			0 (0)
Atherosclerosis coronary syndrome No (%)	54 (33.8)	45 (36)	9 (26.5)	0 (0)		0.42	0 (0)
Drugs, number (%)							
Beta blockers	62 (39)	47 (37)	15 (44)	0 (0)			0 (0)
Central alpha-adrenergic agonists	26 (16)	21 (17)	5 (15)	0 (0)			0 (0)
Sartans	32 (20)	27 (21)	5 (15)	0 (0)			0 (0)
Calcium-channel blockers	47 (29)	38 (30)	9 (26)	0 (0)			0 (0)
ACE inhibitors	66 (41)	55 (43)	11 (32)	21 (16)			0 (0)
Antidiabetic drugs	19 (12)	13 (10)	6 (18)	16 (13)			0 (0)
Antiaggregant drugs	51 (32)	44 (34)	7 (21)	40 (31)			0 (0)
Antidislipidemic drugs	36 (22)	30 (24)	6 (18)	0 (0)			0 (0)
Diuretics	36 (22)	24 (19)	12 (36)	40 (31)			0 (0)

**Table 2:** Allele frequencies of rs4986790 (+896A/G) TLR4, rs333 (Δ32) CCR5 deletion, rs2070744 (−786T/C) eNOs, rs1799752 (D/I) ACE, rs3918242 (−1562C/T) MMP-9, and rs2285053 (−735C/T) MMP-2 SNPs in 161 S-TAA patients and 128 matched controls (2 × 2 comparisons between the different groups with odd ratio (OR) and 95% confidence interval).

Candidate genes	Reference SNP number	Alleles	Patients (*N* = 161)		Matched controls (*N* = 128)		*P* (2 × 2 tables)	OR (95% CI)
TLR4	rs4986790	+896A	321	99.7%	245	96%	0.0008	14.4 (18.1–112.4) *P* = 0.0008
		+896G	1	0.3%	11	4%		

CCR5	rs333	WT	317	98%	238	93%	0.001	4.7 (1.7–13.1) *P* = 0.001

eNOS	rs2070744	Δ32	5	2%	18	7%	0.00007	2.2 (1.5–3.2) *P* < 0.0001
		−786T	207	64%	204	80%		
		−786C	115	36%	52	20%		

ACE	rs1799752	I	125	39%	141	55%	0.0001	1.9 (1.3–2.6) *P* = 0.0001
		D	197	61%	115	45%		

MMP-9	rs3918242	−1562C	282	88%	241	94%	0.011	2.27 (1.2–4.22) *P* = 0.01
		−1562T	40	12%	15	6%		

MMP-2	rs2285053	−735C	287	89%	251	98%	0.00005	6.1 (2.3–15.8) *P* < 0.0001
		−735T	35	11%	5	2%		

All genotypes were in Hardy-Weinberg equilibrium.

**Table 3:** Frequency of +896ATLR4/DACE/−1562TMMP-9/−735TMMP-2 “high responder” (proinflammatory) genotype between patients and controls (2 × 2 comparisons between the different groups with odd ratio (OR) and 95% confidence interval).

Subjects	+896ATLR4/DACE/−1562TMMP-9/−735TMMP-2 “high responder”	Other genotypes	*P* (2 × 2 Table)	OR (95% CI)
Patients (*N* = 161)	46	115	**P** = 0.000009	**4.7 (2.7–9.8) ** **P** < 0.0001
Controls (*N* = 128)	**10**	**118**		

**Table 4:** Systemic plasma mediator's levels “AASSP” from patients and controls.

Systemic mediators examined	Patients (*N* = 161)	Controls (*N* = 128)	*P* values∗
IL-6 (pg/mL)	13.69 ± 2.1	5.1 ± 1.9	<0.0001
TNF-*α* (pg/mL)	16.34 ± 1.2	8.1 ± 2.4	<0.0001
CRP (mg/L)	16.86 ± 2.2	5.6 ± 1.3	<0.0001
MMP-2 (ng/mL)	57.5 ± 2.8	13.54 ± 1.24	<0.0001
MMP-9 (ng/mL)	59.8 ± 2.5	12.7 ± 1.6	<0.0001

Systemic mediators examined	Patients with high responder genotype (*N* = 46)	Patients with other genotypes (*N* = 115)	*P* values∗

IL-6 (pg/mL)	17.66 ± 2.1	9.1 ± 0.9	<0.001
TNF-*α* (pg/mL)	16.78 ± 1.2	10.1 ± 2.2	<0.01
CRP (mg/L)	20.13 ± 1.7	12.1 ± 0.5	0.01
MMP-2 (ng/mL)	61.8 ± 3.8	26.54 ± 1.6	<0.0001
MMP-9 (ng/mL)	59.7 ± 3.7	21.7 ± 2.6	<0.0001

Systemic mediators examined	Patients with high responder genotype (*N* = 46)	Controls with high responder genotype (*N* = 10)	*P* values∗

IL-6 (pg/mL)	17.66 ± 2.1	8.66 ± 2.1	<0.0001
TNF-*α* (pg/mL)	16.78 ± 1.2	10.78 ± 1.2	<0.01
CRP (mg/L)	20.13 ± 1.7	6.13 ± 1.7	<0.0001
MMP-2 (ng/mL)	61.8 ± 3.8	18.8 ± 3.9	<0.0001
MMP-9 (ng/mL)	59.7 ± 3.7	12.7 ± 2.7	<0.0001

*By unpaired *t*-test with Welch correction.

**Table 5:** Comparison of systemic inflammatory mediator's levels “AASSP” from controls bearing combined risk genotype versus controls with other genotypes and between controls bearing +896A TLR4 allele versus controls with +896G TLR4 allele.

Systemic mediators examined	Controls with combined risk genotype (*N* = 10)	Controls with other genotypes (*N* = 118)	*P* values∗
IL-6 (pg/mL)	8.66 ± 2.1	1.1 ± 0.69	<0.0001
TNF-*α* (pg/mL)	10.78 ± 1.2	2.1 ± 1.2	<0.0001
CRP (mg/L)	6.13 ± 1.7	0.9 ± 1.5	<0.0001
MMP-2 (ng/mL)	18.8 ± 3.9	2.54 ± 1.3	<0.0001
MMP-9 (ng/mL)	12.7 ± 2.7	1.7 ± 1.6	<0.0001

Systemic mediators examined	Controls with +896ATLR4 allele (*N* = 10)	Controls with +896G TLR4 allele (*N* = 118)	*P* values∗

IL-6 (pg/mL)	5.1 ± 0.9	0.9 ± 0.5	<0.0001
TNF-*α* (pg/mL)	7.1 ± 0.6	2.1 ± 1.2	<0.001
CRP (mg/L)	4.3 ± 1.1	0.3 ± 1.9	<0.0001
MMP-2 (ng/mL)	10.6 ± 1.8	1.8 ± 0.9	<0.0001
MMP-9 (ng/mL)	8.3 ± 0.7	0.98 ± 1.5	<0.0001

*By unpaired *t*-test with Welch correction.

**Table 6:** Correlations between the number of CD3+CD4+CD8+ CD68+CD20+ cells observed in aorta samples from patients bearing combined risk genotype and the histological abnormalities observed through histopathological and immunohistochemical assays and Tunel testing and levels of IL-6, TNF-*α*, CRP, and MMP-2 and -9.

Variables	Correlations	*P* values∗
Medionecrosis of grade III	0.278	0.02
Cystic-medial change of grade III	0.346	0.001
Elastic fragmentation of grade III	0.467	0.0001
Plurifocal Medial apoptosis	0.333	0.001
Elevated MMP-9 amounts	0.379	0.001
IL-6	0.379	0.003
TNF-*α*	0.445	0.001
CRP	0.467	0.002
MMP-2	0.578	0.001
MMP-9	0.502	0.001

*By nonparametrical Spearman correlation test.

## Nonstandard Abbreviations and Acronyms

AASSP: Arterial-associated senescence secretor phenotype
CT: Computerized tomography
ECHO: Echocardiography
MRI: Magnetic resonance imaging
SNPs: Single nucleotide polymorphisms
TAAD: Stanford type A aortic dissection
TRF: Terminal restriction fragment.

## References

[1] Karavidas A, Lazaros G, Tsiachris D, Pyrgakis V (2010). Aging and the cardiovascular system. *Hellenic Journal of Cardiology*.

[2] Ungvari Z, Kaley G, De Cabo R, Sonntag WE, Csiszar A (2010). Mechanisms of vascular aging: new perspectives. *Journals of Gerontology A Biological Sciences and Medical Sciences*.

[3] Safar ME (2010). Arterial aging-hemodynamic changes and therapeutic options. *Nature Reviews Cardiology*.

[4] Kovacic JC, Moreno P, Nabel EG, Hachinski V, Fuster V (2011). Cellular senescence, vascular disease, and aging: part 2 of a 2-part review: clinical vascular disease in the elderly. *Circulation*.

[5] Maruyama Y (2012). Aging and arterial-cardiac interactions in the elderly. *International Journal of Cardiology*.

[6] Sawabe M (2010). Vascular aging: from molecular mechanism to clinical significance. *Geriatrics and Gerontology International*.

[7] Centers for Disease Control and Prevention WISQARS leading causes of death reports, United States. http://webappa.cdc.gov/cgi-bin/broker.exe.

[8] (2013). *Demographic Population Data*.

[9] Clouse WD, Hallett JW, Schaff HV (2004). Acute aortic dissection: population-based incidence compared with degenerative aortic aneurysm rupture. *Mayo Clinic Proceedings*.

[10] Acosta S, Ögren M, Bengtsson H, Bergqvist D, Lindblad B, Zdanowski Z (2006). Increasing incidence of ruptured abdominal aortic aneurysm: A population-based study. *Journal of Vascular Surgery*.

[11] Aronow WS, Fleg JL, Pepine CJ (2011). ACCF/AHA 2011 expert consensus document on hypertension in the elderly: a report of the American College of Cardiology Foundation Task Force on Clinical Expert Consensus Documents. *Circulation*.

[12] Elefteriades JA, Farkas EA (2010). Thoracic aortic aneurysm clinically pertinent controversies and uncertainties. *Journal of the American College of Cardiology*.

[13] Kuivaniemi H, Platsoucas CD, Tilson MD (2008). Aortic aneurysms: an immune disease with a strong genetic component. *Circulation*.

[14] He R, Guo DC, Sun W (2008). Characterization of the inflammatory cells in ascending thoracic aortic aneurysms in patients with Marfan syndrome, familial thoracic aortic aneurysms, and sporadic aneurysms. *Journal of Thoracic and Cardiovascular Surgery*.

[15] He R, Guo D, Estrera AL (2006). Characterization of the inflammatory and apoptotic cells in the aortas of patients with ascending thoracic aortic aneurysms and dissections. *Journal of Thoracic and Cardiovascular Surgery*.

[16] Balistreri CR, Pisano C, D'Amico T (2013). The role of inflammation in type A aortic dissection: a pilot study. *European Journal of Inflammation*.

[17] Zhang X, Shen YH, LeMaire SA (2009). Thoracic aortic dissection: are matrix metalloproteinases involved?. *Vascular*.

[18] Hiratzka,L. F., Bakris GL, Beckman JA (2010). 2010 ACCF/AHA/AATS/ACR/ASA/SCA/SCAI/SIR/STS/SVM guidelines for the diagnosis and management of patients with thoracic aortic disease: a report of the American College of Cardiology Foundation/American Heart Association Task Force on Practice Guidelines, American Association for Thoracic Surgery, American College of Radiology, American Stroke Association, Society of Cardiovascular Anesthesiologists , Society for Cardiovascular Angiography and Interventions, Society of Interventional Radiology, Society of Thoracic Surgeons, and Society for Vascular Medicine. *Journal of American College of Cardiology*.

[19] El Assar M, Angulo J, Rodríguez-Mañas L (2013). Oxidative stress and vascular inflammation in aging. *Free Radical Biology Medicine*.

[20] Bachschmid MM, Schildknecht S, Matsui R (2013). Vascular aging: chronic oxidative stress and impairment of redox signaling. Consequences for vascular homeostasis and disease. *Annals of Medicine*.

[21] Csiszar A, Wang M, Lakatta EG, Ungvari Z (2008). Inflammation and endothelial dysfunction during aging: role of NF-*κ*B. *Journal of Applied Physiology*.

[22] Matzinger P (2002). The danger model: a renewed sense of self. *Science*.

[23] Pryshchep O, Ma-Krupa W, Younge BR, Goronzy JJ, Weyand CM (2008). Vessel-specific toll-like receptor profiles in human medium and large arteries. *Circulation*.

[24] Sirisinha S (2011). Insight into the mechanisms regulating immunehomeostasis in health and disease. *Asian Pacific Journal of Allergy and Immunology*.

[25] Hollestelle SCG, De Vries MR, Van Keulen JK (2004). Toll-like receptor 4 is involved in outward arterial remodeling. *Circulation*.

[26] Vink A, de Kleijn DPV, Pasterkamp G (2004). Functional role for toll-like receptors in atherosclerosis and arterial remodeling. *Current Opinion in Lipidology*.

[27] Birukov KG (2009). Cyclic stretch, reactive oxygen species, and vascular remodeling. *Antioxidants and Redox Signaling*.

[28] Wang M, Monticone RE, Lakatta EG (2010). Arterial aging: a journey into subclinical arterial disease. *Current Opinion in Nephrology and Hypertension*.

[29] Balistreri CR, Candore G, Accardi G, Colonna-Romano G, Lio D (2013). NF-*κ*B pathway activators as potential ageing biomarkers: targets for new therapeutic strategies. *Immunity and Ageing*.

[30] Balistreri CR, Candore G, Colonna-Romano G (2004). Role of toll-like receptor 4 in acute myocardial infarction and longevity. *Journal of the American Medical Association*.

[31] Frantz S, Ertl G, Bauersachs J (2007). Mechanisms of disease: toll-like receptors in cardiovascular disease. *Nature Clinical Practice Cardiovascular Medicine*.

[32] Balistreri CR, Colonna-Romano G, Lio D, Candore G, Caruso C (2009). TLR4 polymorphisms and ageing: Implications for the pathophysiology of age-related diseases. *Journal of Clinical Immunology*.

[33] Ionita MG, Arslan F, de Kleijn DPV, Pasterkamp G (2010). Endogenous inflammatory molecules engage toll-like receptors in cardiovascular disease. *Journal of Innate Immunity*.

[34] Hofmann U, Ertl G, Frantz S (2011). Toll-like receptors as potential therapeutic targets in cardiac dysfunction. *Expert Opinion on Therapeutic Targets*.

[35] Navi A, Patel H, Shaw S, Baker D, Tsui J (2013). Therapeutic role of toll-like receptor modification in cardiovascular dysfunction. *Vascular Pharmacology*.

[36] Incalcaterra E, Accardi G, Balistreri CR (2013). Pro-inflammatory genetic markers of atherosclerosis topical collection on genetics. *Current Atherosclerosis Reports*.

[37] Song Y, Shen H, Schenten D, Shan P, Lee PJ, Goldstein DR (2012). Aging enhances the basal production of IL-6 and CCL2 in vascular smooth muscle cells. *Arteriosclerosis, Thrombosis, and Vascular Biology*.

[38] Eissler R, Schmaderer C, Rusai K (2011). Hypertension augments cardiac Toll-like receptor 4 expression and activity. *Hypertension Research*.

[39] González-Ramos M, Calleros L, López-Ongil S (2013). HSP70 increases extracellular matrix production by human vascular smooth muscle through TGF-beta1 up-regulation. *International Journal of Biochemistry and Cell Biology*.

[40] Li H, Xu H, Liu S (2011). Toll-like receptors 4 induces expression of matrix metalloproteinase-9 in human aortic smooth muscle cells. *Molecular Biology Reports*.

[41] Bucci M, Vellecco V, Harrington L (2013). Cross-talk between toll-like receptor 4 (TLR4) and proteinase-activated receptor 2 (PAR2) is involved in vascular function. *The British Journal of Pharmacology*.

[42] Pirianov G, Torsney E, Howe F, Cockerill GW (2012). Rosiglitazone negatively regulates c-Jun N-terminal kinase and toll-like receptor 4 proinflammatory signalling during initiation of experimental aortic aneurysms. *Atherosclerosis*.

[43] Balistreri CR, Bonfigli AR, Boemi M (2014). Evidences of +896 A/G TLR4 polymorphism as an indicative of prevalence of complications in T2DM patients. *Mediators of Inflammation*.

[44] Ono M, Goerler H, Boethig D, Westhoff-Bleck M, Breymann T (2009). Current surgical management of ascending aortic aneurysm in children and young adults. *Annals of Thoracic Surgery*.

[45] David TE (2010). Surgical treatment of ascending aorta and aortic root aneurysms. *Progress in Cardiovascular Diseases*.

[46] Pisano C, Maresi E, Balistreri CR (2012). Histological and genetic studies in patients with bicuspid aortic valve and ascending aorta complications. *Interactive Cardiovascular and Thoracic Surgery*.

[47] Pisano C, Maresi E, Merlo D (2012). A particular phenotype of ascending aorta aneurysms as precursor of type A aortic dissection. *Interactive Cardiovascular and Thoracic Surgery*.

[48] Balistreri CR, Pisano C, Candore G, Maresi E, Codispoti M, Ruvolo G (2013). Focus on the unique mechanisms involved in thoracic aortic aneurysm formation in bicuspid aortic valve versus tricuspid aortic valve patients: clinical implications of a pilot study. *European Journal of Cardio-thoracic Surgery*.

[49] Balistreri CR, Maresi E, Pisano C (2014). Identification of three particular morphological phenotypes in sporadic thoracic aortic aneurysm (S-TAA): the phenotype III as S-TAA biomarker in aged individuals. *Rejuvenation Research*.

[50] Bechtel JFM, Noack F, Sayk F, Erasmi AW, Bartels C, Sievers H (2003). Histopathological grading of ascending aortic aneurysm: comparison of patients with bicuspid versus tricuspid aortic valve. *Journal of Heart Valve Disease*.

[51] Balistreri CR, Pisano C, Merlo D (2012). Is the mean blood leukocyte telomere length a predictor for sporadic thoracic aortic aneurysm? Data from a preliminary study. *Rejuvenation Research*.

[52] de Meyer T, Rietzschel ER, de Buyzere ML, van Criekinge W, Bekaert S (2011). Telomere length and cardiovascular aging: the means to the ends?. *Ageing Research Reviews*.

[53] Fyhrquist F, Saijonmaa O (2012). Telomere length and cardiovascular aging. *Annals of Medicine*.

[54] Balistreri CR, Caruso C, Listì F, Colonna-Romano G, Lio D, Candore G (2011). LPS-mediated production of pro/anti-inflammatory cytokines and eicosanoids in whole blood samples: Biological effects of +896A/G TLR4 polymorphism in a Sicilian population of healthy subjects. *Mechanisms of Ageing and Development*.

[55] Balistreri CR, Candore G, Accardi G (2012). Genetics of longevity. Data from the studies on Sicilian centenarians. *Immunity and Ageing*.

[56] Balistreri CR, Grimaldi MP, Chiappelli M (2008). Association between the polymorphisms of TLR4 and CD14 genes and Alzheimer's disease. *Current Pharmaceutical Design*.

[57] Balistreri CR, Caruso C, Carruba G (2010). A pilot study on prostate cancer risk and pro-inflammatory genotypes: pathophysiology and therapeutic implications. *Current Pharmaceutical Design*.

[58] Pompilio G, Capogrossi MC, Pesce M (2009). Endothelial progenitor cells and cardiovascular homeostasis: clinical implications. *International Journal of Cardiology*.

[59] Richardson MR, Yoder MC (2011). Endothelial progenitor cells: quo vadis?. *The Journal of Molecular and Cellular Cardiology*.

[60] Toya SP, Malik AB (2012). Role of endothelial injury in disease mechanisms and contribution of progenitor cells in mediating endothelial repair. *Immunobiology*.

[61] He J, Xiao Z, Chen X (2010). The expression of functional toll-like receptor 4 is associated with proliferation and maintenance of stem cell phenotype in endothelial progenitor cells (EPCs). *Journal of Cellular Biochemistry*.

[62] Olivieri F, Rippo MR, Prattichizzo F (2013). Toll like receptor signaling in “inflammaging”: MicroRNA as new players. *Immunity and Ageing*.

[63] Lin CP, Lin FY, Huang PH (2013). Endothelial progenitor cell dysfunction in cardiovascular diseases: role of reactive oxygen species and inflammation. *BioMed Research International*.

[64] Li Q, Han J, Chen H, Mo X (2013). Reduced circulating endothelial progenitor cells in the coronary slow flow phenomenon. *Coronary Artery Disease*.

[65] Feng Y, Yang S, Xiao B (2010). Decreased in the number and function of circulation endothelial progenitor cells in patients with avascular necrosis of the femoral head. *Bone*.

