# Supplementary material for: Can the TLR-4-Mediated Signaling Pathway Be “A Key Inflammatory Promoter for Sporadic TAA”?
Source: Mediators Inflamm. 2014 Jul 10;2014:349476. doi: 10.1155/2014/349476 (PMC4120489; doi:10.1155/2014/349476)
Supplement: Supplementary file 1 — In this section, we reported in detail the procedure used for evaluating aortic transverse diameter sizes. In addition, criteria, definitions and grading systems for tissue sample collection, staining, histopathological and immunohistochemical assessment, ELISA for quantifying inflammatory plasma molecules, Terminal deoxynucleotidyl transferase dUTP Nick End Labeling (TUNEL) testing for evaluating apoptosis, semiquantitative MMP-9 evaluation, assessment of mean terminal restriction fragment length and genotyping were also described in detail. Furthermore, we reported in Table 1S information about the 6 genes and 10 SNPs analysed and their Biological Effects. In Figures 1S and 2S we reported the control aortas and histo-pathological abnormalities in aorta tissues of S-TAA patients and medial apoptosis and MMP-9 amounts in tissue samples, reciprocally. [file 349476.f1.pdf]

Supplementary material:

Evaluation of aortic transverse diameter sizes

Their evaluation was made both in operating room and preoperatively by ECHO and CT estimations and performed as follows: estimating dimensions of aortic annulus, sinuses of Valsalva and proximal ascending aorta (above 2,5 cm of the sinotubular junction) in parasternal long-axis; evaluating those of aortic arch from suprasternal view; detecting other measurements perpendicular to aorta long axis using leading edge to leading edge method [1S] Color Doppler was used to assess presence and severity of aortic regurgitation, and aortic stenosis was excluded both by pulsed-wave and continuous-wave Doppler. Furthermore, aortic root and ascending aorta diameter sizes were carried out similarly to helical CT image analysis technique, described by Hager et al [2S]. The aorta was, thus, sized at 3 levels: level 1, aortic sinuses; level 2, sinotubular junction; level 3, right main pulmonary artery. Slices were adjusted for each level to obtain an oblique axial plane perpendicular to aorta course. Internal aorta diameter was then measured with electronic calipers in 2 directions at 90° to one another. The mean of these values was deemed diameter to be used for calculations.

Aortic specimens, histopathological assays and identification of three phenotypes

Because of histological condition of some patient aortas, full aortic segments were unfortunately collected from resected aortic wall of only 100 S-TAA patients at surgical time. They were fixed in 10% neutral buffered formalin for 24 hours and then processed for routine paraffin embedding. Surgical specimens subsequently were photographer and measured (maximum transverse diameter). For microscopic examinations, multiple histological sections from each sample were prepared and stained with hematoxylin-eosin, Weigert-van Gieson, Alcian-PAS (see Figure 1S.)

Using the grading systems defined by Matthias Bechtel and colleagues [31] (see Figure 1S and 2S in online Supplementary Material) and previously described in our recent studies[16, 27-31], we detected three morphological MD phenotypes. They were characterized to have a

different quantitative relationship of cystic MD, fibrosis, apoptosis and amount of MMP-9 (see Figure 1S and 2S), described as follows and previously reported in a previous study[30]

Phenotype I: cystic medial degeneration balanced by a substitutive fibrosis, in absence of medial apoptosis and with a low MMP-9 concentration;

Phenotype II: higher cystic medial degeneration than substitutive fibrosis, with focal medial apoptosis, and with mainly a modest MMP-9 amount;

Phenotype III: elevated cystic medial degeneration, without substitutive fibrosis, with plurifocal medial apoptosis, and with an elevated MMP-9 concentration.

Immunohystochemical assays

Immunohistochemical analyses were performed on 5µm-thick paraffin-embedded sections. Deparaffinized sections were treated for 20 min in a microwave oven in 10 mM citrate buffer PH6, or TRIS/EDTA PH9. Sections were then incubated for 1 h with specific monoclonal antibodies (Ab)s against CD3 (Clone LN10, NCL-L-CD3, clone PS1, Novocastra Laboratories Ltd, UK 1:100), CD45 (Santa Cruz, Biotechnology, Inc, Santa Cruz, Calif. 1:100), CD4 Ab-2 (Clone 1 F6, NeoMarkers, InC USA, 1:10), CD8 Ab-1 (Clone C8/144B, NCL-L-CD8 295 mouse Novocastra Laboratories Ldt, U.K. 1:50), CD20 (clone L 26, Dako Italia Spa 1:50)), CD68 (clone PG-M1, Dako Italia SpA 1:50), MMP-9 (Clone 15W2, NCL-MMP9 439, Novocastra Laboratories Ltd, UK 1:50), or isotype-matched controls at appropriate dilutions. After washing in TBS 1X (Tris-buffered solution), staining was performed by biotinylated link antibody and streptovidin labeled with PEROXIDASE KITS (Dako, North America, Inc, USA) and was detected using AEC (3-amino-9-ethylcarbazole) substrate chromogen. After counterstaining of cells and tissue sections was performed using aqueous hematoxylin (Novocastra Laboratories Ldt, U.K) (see Figure 2S).

Inflammatory and immune cells were counted in 10 contiguous high-power fields (magnification 400x) under an Olympus fluorescent microscope (America Inc, Melville, NY) by two independent observers.

Tunel Testing

We performed TdT (Terminal deoxynucleotidyl Transferase)-mediated X-dUTP (deoxyuridine triphosphate nucleotides) nick end-labeling (TUNEL) reaction (“In situ cell death detection kit”, Roche Diagnostics S.p.A, Milano, Italy) on full-thickness aortic wall paraffin sections (5 µm). Tissue were deparaffined and then permeablised with PBS 0,1% sodium citrate/0,1% Triton X-100. Specimens were then incubated with TdT and fluorescein-labeled dUTP in a humidified atmosphere for 1 h at 37°C. In situ apoptosis staining was revealed by using an AP converter. DNA strand breaks were detected by using the 5-bromo-4chloro-3-indolyl-phosphate (BCIP/NBT, Dako, Italy) substrate chromogen. Tissues were subsequently counterstained with eosin under light microscopy (see Figure 2S) .

Genotyping

DNA samples of 128 matched controls and 161 S-TAA cases were extracted from peripheral blood samples collected in tripotassium EDTA and purified by using a QIAamp Blood DNA Maxi kit (Qiagen, Dusseldorf, Germany). Samples were genotyped for ten SNPs located in promoter and coding regions of selected genes (see Table S1**)**. Two procedures were utilised, such as Restriction Fragment Length Polymorphism-PCR (RFLP-PCR) and single specific primer (SSP)-PCR. For genotyping +896A/G (Asp299Gly, rs4986790), +1196C/T (Thr399Ile, rs4986791) TLR4 SNPs and Δ32 CCR5 deletions (rs333) procedures previously described were used [3S, 4S].

For identification of -1562C/T MMP9 (rs3918242), -1306C/T (rs243865) and -735C/T MMP2 (no rs available designation) SNPs, RFLP-PCR procedure was used, followed by restriction cleavage with ShpI, AccI and Hinf I (New England Biolabs, USA) respectively, and separation of DNA fragments by electrophoresis [5S]. For genotyping +894G/T (Glu208Asp, rs 1799983) and -786T/C (rs2070744) SNPs in eNOs gene, RFLP-PCR was also executed, followed by restriction cleavage with Ban I and Nae I (New England Biolabs, USA) respectively, and separation of DNA fragments by electrophoresis [5S, 6S].A SSP-PCR was, while, assessed for genotyping 4a/4b eNOs and D/I (rs1799752) ACE SNPs [6-8S].

Inflammatory plasma molecule measurements

Venous blood samples were collected from all patients and control subjects in a fasting state (more than 8 h without food administration from onset). In patients who underwent surgical treatment, blood samples were collected before surgery. Plasma samples were obtained after a centrifugation of 3500 rpm at 4°C for 15 min immediately after collection and then stored at −80 °C for further analysis. Plasma IL-6, TNF-α, MMP-2 and MMP-9 were measured by using ELISA technique, with the R&D Systems (Minneapolis, MN, USA), according to the manufacturer's instructions. CRP was determined by a high-sensitivity assay using a BN II nephelometer (Dade Behring, Marburg, Germany). Detection limits were 0.7 pg/ml, 0.5 pg/ml, 0.154 ng/ml, 0.156 ng/ml and 0.17mg/l for IL-6, TNF-α, MMP-2, MMP-9 and CRP respectively. All assays were run in duplicate.

Assessment of mean terminal restriction fragment length

Genomic DNA was extracted from leukocytes of both the two cohorts and the mean terminal restriction fragment (TRF) length, a marker of telomere length, was measured by a chemiluminesence technique using TAGGG telomere length assay kits (Roche-Applied Science, Germany) [9S]. In the present study, we examined the mean TRF length in 30 patients and 30 controls selected randomly, but having the same age and gender. Accordingly, the same number of males and females (20 and 10, respectively) characterised both the two cohorts studied.

Table S1. Genes (accession number), SNPs (accession number), and substitutions investigated in the study

Genes	SNPs	Biological Effect
TLR4 (NM-138554.1)	+896A/G (Asp299Gly; rs4986790)<br><br>+1196C/T (Thr 399Ile; rs4986791)	Determining a single amino acid substitution in the extracellular receptor domain and, hence, a blunted innate/inflammatory response to both foreign pathogens and endogenously generated inflammatory ligands [10,11S]
CCR5 (NM-00579)	Deletion 32 (rs333)	A non functional allele resulting from a 32-bp deletion in exon 4 (CCR5_32), determines a loss of expression of functional CCR5 receptor [12S]
MMP9 (NM-004985)	-1562C/T (rs3918242)	Located within a putative binding site for a regulator factor of the gene transcription, determining an increased expression of MMP-9 enzyme[5S]
MMP2 (NM-001121363.1)	-1306C/T (rs243865)<br><br>-735C/T (rs 2285053)	They are located within a putative binding site for a regulator factor, Sp1, of the gene transcription, determining an increased expression of MMP2- enzyme [5S]
eNOs (NM-000594)	894G/T (Glu208Asp ; rs 1799983)	Determining a single amino acid substitution (Glu /Asp -208), associated with a reduced basal production of NO and an increased amount of plasmatic homocysteine[6S,7S]
	-786T/C (rs2070744)	SNP located in the promoter region determining a reduced gene transcription[6S,7S]
	4a/4b VNTR repeat	A VNTR repeat of 27 bp in a intron region associated with an altered production of NO[6S,7S]
ACE (NM-152830.1)	D/I SNP (rs1799752)	Determining changes in the plasmatic and cellular concentration of ACE enzyme. The D allele is associated with a high risk and susceptibility of vascular disorders[8S]

Supplementary References:

1S. Evangelista A, Flachskampt FA, Erbe R , et al. Echocardiography in aortic diseases: EAE recommendations for clinical practice. *Eur J Echocardiogr*. 2010; 11: 645-58.

2S. Hager A, Kaemmerer H, Rapp-Bernhardt I, et al. Diameters of the thoracic aorta throughout life measured with helical computed tomography. *J Thorac Cardiovasc Surg* 2002;123:1060–1066.

3S. Balistreri CR, Candore G, Lio D, Candore G, Caruso C. Role of TLR4 receptor polymorphisms in Boutonneuse fever. *Int J Immunopathol Pharmacol* 2005; 18: 655-660.

4S. Balistreri CR, Grimaldi MP, Vasto S, Listi F, Chiappelli M, Licastro F, Lio D, Caruso C, Candore G.et al. Association between the polymorphism of CCR5 and Alzheimer's disease: results of a study performed on male and female patients from Northern Italy. *Ann N Y Acad Sci.* 2006;1089:454-61.

5S. Gremlich S, Nguyen D, Reymondin D, et al. Fetal MMP2/MMP9 polymorphisms and intrauterine growth restriction risk. *J Reprod Immunol*. 2007;74:143-151.

6S. Fatini C, Sofi F, Sticchi E, et al. eNOS G894T polymorphism as a mild predisposing factor for abdominal aortic aneurysm. *J Vasc Surg*. 2005;42:415-419.

7S. Tang FY, Liu FY, Xie XW. Association of angiotensin-converting enzyme and endothelial Nitric Oxide synthase gene polymorphisms with vascular disease in ESRD patients in a Chinese population. *Mol Cell Biochem.* 2008;319:33-39.

8S. Fatini C, Pratesi G, Sofi F, et al. ACE DD genotype: a predisposing factor for abdominal aortic aneurysm. Eur J Vasc Endovasc Surg. 2005;29:227-232.

9S. Balistreri CR, Pisano C, Merlo D, Fattouch K, Caruso M, Incalcaterra E, Colonna-Romano G, Candore G. Is the mean blood leukocyte telomere length a predictor for sporadic thoracic aortic aneurysm? Data from a preliminary study. *Rejuvenation Res*. 2012;15:170-3.

10S. Balistreri CR, Colonna-Romano G, Lio D, Candore G, Caruso C. TLR4 polymorphisms and ageing: implications for the pathophysiology of age-related diseases. *J Clin Immunol*. 2009;29:406-15.

11S. Balistreri CR, Caruso C, Listì F, Colonna-Romano G, Lio D, Candore G. LPS-mediated production of pro/anti-inflammatory cytokines and eicosanoids in whole blood samples: biological effects of +896A/G TLR4 polymorphism in a Sicilian population of healthy subjects. *Mech Ageing Dev*. 2011;132:86-92.

12S. Balistreri CR, Caruso C, Grimaldi MP, Listì F, Vasto S, Orlando V, Campagna AM, Lio D, Candore G. CCR5 receptor: biologic and genetic implications in age-related diseases. *Ann N Y Acad Sci.* 2007;1100:162-72.

Legends of figures

Figure 1S. Control aortas and histo-pathological abnormalities in aorta tissues of S-TAA patients. Normal aorta (**a, a1, a2, a3**). **Cystic medial** changes of grade I (**b**), II (**c**) and III (**d**); **Elastic fragmentation** of grade I (**b1**), II (**c1**) and III (**d1**). **Medial fibrosis** of grade I (**b2**), II (**c2) and** III (**d2**). **Medial necrosis** of grade I (**b3**), II (**c3**) and III (**d3**).

Figure 2S. Medial apoptosis and MMP-9 amounts in tissue samples. In a2 and a3 images of control aorta wall conditions. In b2 and c2 images of focal and plurifocal medial apoptosis, respectively, in patient tissues. In b3 (low), c3 (moderate) and d3 (elevated) levels of MMP-9 in patient samples

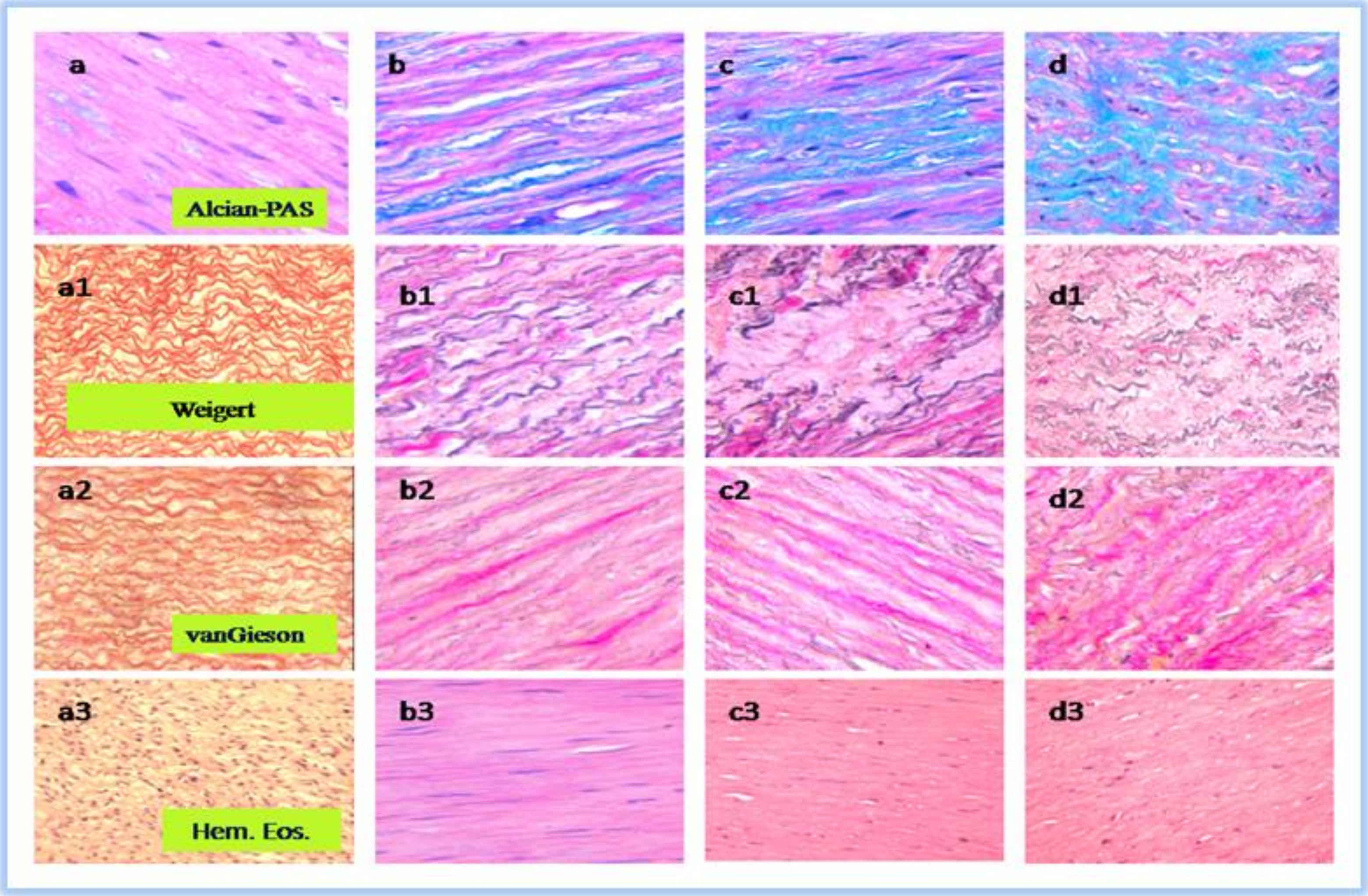

Fig. 1S. Control aortas and histo-pathological abnormalities in aorta tissues of S-TAA patients.
Normal aorta (**a, a1, a2, a3**). **Cystic medial** changes of grade I (**b**), II (**c**) and III (**d**); **Elastic fragmentation** of grade I (**b1**), II (**c1**) and III (**d1**). **Medial fibrosis** of grade I (**b2**), II (**c2**) and III (**d2**). **Medial necrosis** of grade I (**b3**), II (**c3**) and III (**d3**).

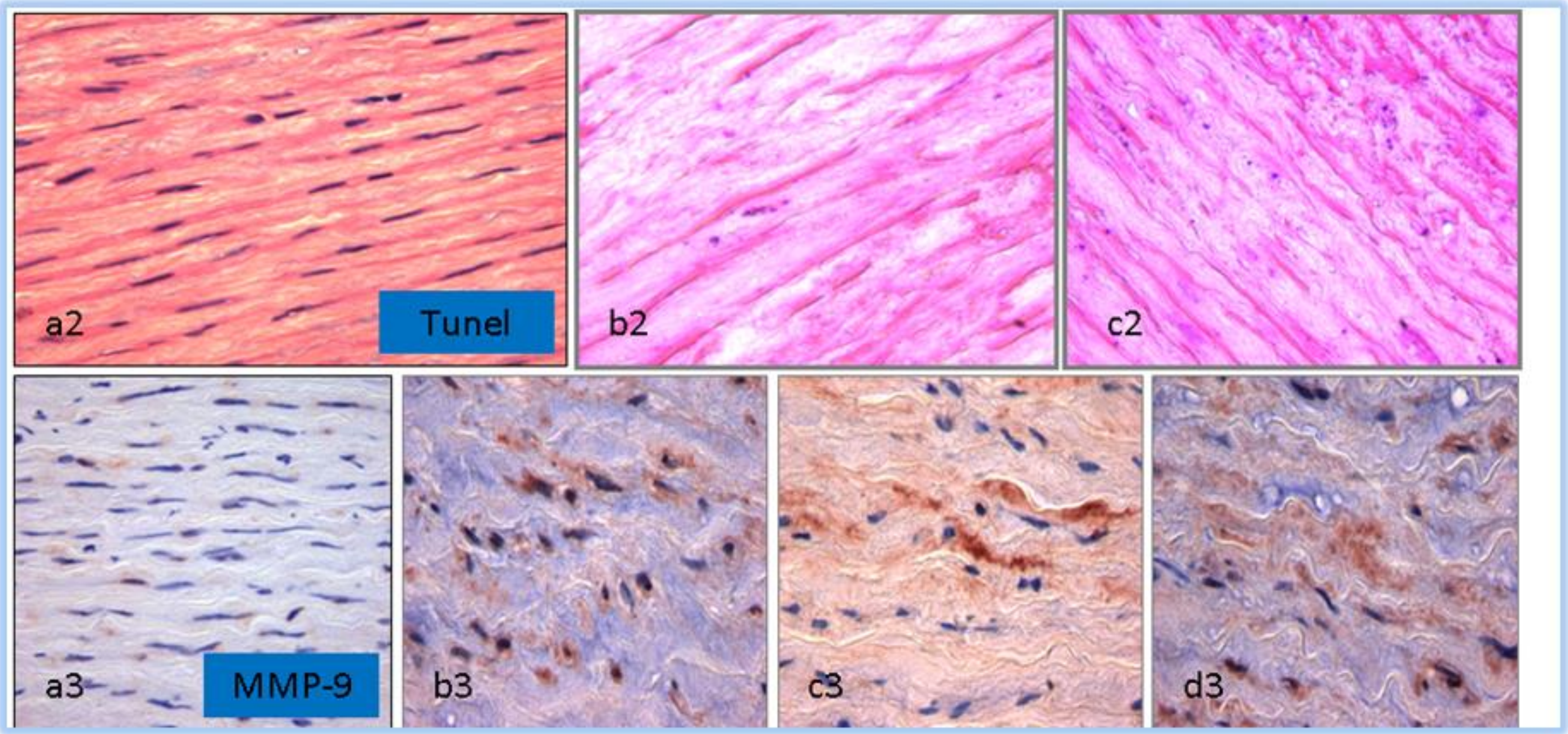

Fig.2S Medial apoptosis and MMP-9 amounts in tissue samples. In a2 and a3 images of control aorta wall conditions. In b2 and c2 images of focal and plurifocal medial apoptosis, respectively, in patient tissues. In b3 (low), c3 (moderate) and d3 (elevated) levels of MMP-9 in patient samples.
